# Community health education improves child health care in Rural Western China

**DOI:** 10.1186/s12887-018-1084-0

**Published:** 2018-04-10

**Authors:** Weifeng Liang, Yuan Xing, Miaomiao Pang, Duolao Wang, Hong Yan

**Affiliations:** 1 Department of Epidemiology and Health Statistics, School of Public Health, Xi’an Jiaotong University College of Medicine, P.O. Box 46, No.76 West Yanta Road, Xi’an, Shaanxi 710061 People’s Republic of China; 2 Xi’an Center for Disease Control and Prevention, Xi’an, Shaanxi 710054 People’s Republic of China; 3 Shaanxi Provincial Corps Hospital of Chinese People’s Armed Police Force, Xi’an, Shaanxi 710054 People’s Republic of China; 4 Department of Clinical Sciences, Liverpool School of Tropical Medicine, Liverpool, L3 5QA UK

**Keywords:** Rural western China, Child feeding practices, Breastfeeding, Complementary foods, Stunting

## Abstract

**Background:**

Rural infant growth failure has been highlighted as a priority for action in China’s national nutrition and child development policies. The aim of this paper was to evaluate the effect of community-based intervention project on child feeding, child health care and child growth.

**Methods:**

From 2001 to 2005, UNICEF and China’s Ministry of Health worked together to develop holistic strategies for child health care. All the interventions were implemented through the three-tier (county-township-village) rural health care network.In this study, 34 counties were included in both surveys in 2001 and 2005. Among these 34 counties, nine were subjected to the intervention and 25 counties were used as controls. In nine intervention counties, leaflets containing information of supplemental feeding of infants and young children were printed and distributed to women during hospital delivery or visit to newborn by village doctors. Two cross-sectional surveys were both conducted from July to early September in 2001 and 2005. We calculated Z-scores of height-for-age (HAZ), weight-for-age (WAZ) and weight-for-height (WHZ), with the new WHO growth standard. HAZ < − 2 was defined as stunting, WAZ < − 2 was defined as underweight, and WHZ < − 2 was defined as wasting.

**Results:**

Following the four-year study period, the parents in the intervention group showed significantly better infant and young child feeding practices and behaviors of child care than did their control group counterparts. In addition, all three anthropometric indicators in 2005 in the intervention group were better than in the control, with stunting 4.9% lower (*p* < 0.001), underweight 2.2% lower (*p* < 0.001), and wasting 1.0% lower (*p* < 0.05).

**Conclusions:**

We concluded that the health care education intervention embed in government had the potential to be successfully promoted in rural western China.

**Electronic supplementary material:**

The online version of this article (10.1186/s12887-018-1084-0) contains supplementary material, which is available to authorized users.

## Background

Physical growth is often considered as a good global indicator of children’s well-being, because infections and unsatisfactory feeding practices, or more often a combination of the two, are major factors affecting their physical growth and mental development [1]. Many studies have shown an association between increased severity of anthropometric deficits and infant and child mortality, and a substantial contribution to child mortality is made by all degrees of malnutrition [2–4].

Child malnutrition may also have an adverse effect on children’s intellectual development [2]. Child growth retardation is associated with adult life dysfunction [3, 4], decreased ability to work, affecting economic productivity [5].

Child development refers to the orderly appearance of the interdependent skills of sensory, cognitive and social emotional functions. This emergence depends on, and is interlinked with, a child’s good nutrition and health. As “A World Fit for Children” states, “…children should be physically healthy, mentally alert, emotionally secure, socially competent and ready to learn” [6]. The most important years for a child’s survival, growth, and development are prenatally through the transition to school, with the fastest period of growth occurring during the first three to four years of life, when the child’s brain is rapidly growing and adapting to the environment. During this period, the developing brain is most sensitive to the effects of malnutrition, toxins, stress, and lack of nurturing or brain stimulation [7].

A common model of health promotion to improve nutrition and child development has three major components [8]: (i) enhancing nutritional intake (including exclusive breastfeeding up to six months of life, appropriate provision of weaning foods, and micronutrient supplementation); (ii) improving health and sanitation services; and (iii) enhancing the level of child care, such as early childhood stimulation programmes and monitoring children’s growth and development.

Rural infant growth failure was highlighted as a priority for action in China’s national nutrition and child development policies [9]. It was considered important to both enhance the nutritional status of rural children and to find effective ways to improve early childhood development. Thus UNICEF and China’s Ministry of Health developed a wide ranging programme of strategies for child health care intervention, including the “Rural Primary Health Care project (RPHC)” from January 1, 2002 to December 31, 2005.. This study was designed to evaluate the effect of this project on child feeding, child health care and child growth.

## Methods

### Population and setting

The RPHC project was implemented in 10 provinces of rural western China, where there is a total land area of 151,548 km^2^, a population of 19.1 million, and 52 nationalities [10].

A 1999 Chinese national survey in children under three years of age in rural western areas reported the prevalence of stunting was 23.0%, while underweight and wasting were 22.6% and 7.5%, respectively [11].

### Study design

Through a quasi experimental design, changes in anthropometric indices from 2001 to 2005 were evaluated between intervention and control arm. Nine counties were subjected to the intervention and 25 counties were used as controls.

### RPHC intervention

The Rural Primary Health Care project (RPHC) was a province-level government-led integrated intervention project, which targeted children aged less than 3 years. The main objectives were: (i) to promote healthy growth and reduce the incidence of malnutrition in infants and young children through the use of recommended feeding and dietary practices [12], (ii) to monitor child growth and development; (iii) to improve the Hepatitis B vaccination rate, (iv) to improve vitamin A supplementation intake rate among children. All the interventions were implemented through the three-tier “county-township-village” rural health care network, which is the standard channel through which all national health policies are implemented.

The county-level staffs were responsible for policy supervision across the whole county. Township-level staffs were responsible for the hepatitis B vaccination initiative for all the township’s children. Village-level staffs were responsible for vitamin A supplementation supply for all the village’s children, monitoring each child’s growth and provision of appropriate nutrition guidance and health education for parents.

### Data collection

Two cross-sectional surveys were used for data collection, one at the beginning and one at the end of intervention (2001 and 2005, respectively). The two samples were obtained using a stratified multistage cluster random sampling method, taking into account the hierarchical structure of Chinese administrative districts and the imbalanced population distribution between the different provinces. In each county, five townships were selected with probability proportional to total population (from the countries’ demographic data). In each selected township, four villages were selected randomly, also with probability proportional to population size. Finally in each village 16 children less than three years old were randomly selected from all the children aged < 3 years in this village.

All participants were interviewed face-to-face by trained professional interviewers from the Xi’an Jiaotong University College of Medicine. The children’s mothers were interviewed and a questionnaire was used to collect information on the child and family socio-demographic characteristics, child health care, mothers’ feeding/caring practices, hepatitis B vaccination status, and vitamin A supplementation intake. The age of each child was obtained from their Permanent Residence Registration, where birth dates are recorded (Additional files 1 and 2).

### Anthropometric measurements

Lengths were measured in children from birth to 36 completed months using a standard calibrated board (accurate to 1 mm). Weights were measured using portable electronic scales with measurement accuracy to 0.1 kg. All equipment was newly purchased, recommended by UNICEF and calibrated daily.

### Data management and statistical analysis

A database was established using Epi Info Version 6.0 (CDC, Atlanta, GA, USA), and data were double-entered to reduce data entry errors.

We hypothesized that the intervention would lead to improved feeding practices, child care, and growth among children in the areas of the intervention. The main outcomes variables were: (1) Feeding practices (including early initiation of breastfeeding, exclusive breastfeeding, and child feeding index); (2) Child care (including give separate cooking for children, attendance rate at a health service for cold symptoms in two weeks, attendance rate at a health service for diarrhea in two weeks, vitamin A supplementation intake rate, and hepatitis B vaccination rate); (3) Anthropometric status (including stunting, underweight, and wasting).

A household wealth index was constructed from an inventory of five levels of household assets or facilities from both the intervention and control groups for 2001 and 2005, using a principal component analysis method, and this index was categorized as tertiles as indicators of the least, middle, and wealthiest households [13].

Early initiation of breastfeeding was used as an indicator to assess breastfeeding practices [14]. Exclusive breastfeeding of infants up to 6 months old was used to assess the feeding practices of children less than 6 months of age. The child feeding index was used to assess the feeding practices of children over 6 months of age (Table 1) [15, 16].

**Table 1:** Variables and scoring system used to create the child

Variables	Item Score	6–9 month	9–12 month	12–36 month
Breastfeeding	2	No = 0; Yes = 2	No = 0; Yes = 2	No = 0; Yes = 1
Milk and related products				0–2 times/w = 0
				1–4 times/w = 1
				5+ times/w = 2
Cereals	1	0–1 time/d = 0	0–2 times/d = 0	
		1+ time/d = 1	2+ times/d = 1	
Beans and related products	2	0–1 time/m = 0	0–1 time/m = 0	0–1 time/m = 0
		1 time/m-4 times/w = 1	1 time/m-4 times/w = 1	1 time/m-4 times/w = 1
		5+ times/w = 2	5+ times/w = 2	5+ times/w = 2
Egg	2	0–1 time/m = 0	0–1 time/m = 0	0–1 time/m = 0
		1 time/m-4 times/w = 1	1 time/m-4 times/w = 1	1 time/m-4 times/w = 1
		5+ times/w = 2	5+ times/w = 2	5+ times/w = 2
Fish	2	0–1 time/m = 0	0–1 time/m = 0	0–1 time/m = 0
		1 time/m-4 times/w = 1	1 time/m-4 times/w = 1	1 time/m-4 times/w = 1
		5+ times/w = 2	5+ times/w = 2	5+ times/w = 2
Meat	2	0–1 time/m = 0	0–1 time/m = 0	0–1 time/m = 0
		1 time/m-4 times/w = 1	1 time/m-4 times/w = 1	1 time/m-4 times/w = 1
		5+ times/w = 2	5+ times/w = 2	5+ times/w = 2
Dietary diversity	2	0 kind = 0	0 kind = 0	kind = 0
		1–3 types = 1	1–3 types = 1	1–3 types = 1
		4 + types = 2	4+ types = 2	4+ types = 2
Total score		13 points	13 points	13 points

^1^Time/d: times per day; time/w: times per week; time/m: times per month

We calculated Z-scores of height-for-age (HAZ), weight-for-age (WAZ) and weight-for-height (WHZ), with the new WHO growth standard [17]. HAZ < − 2 was defined as stunting, WAZ < − 2 was defined as underweight, and WHZ < − 2 was defined as wasting [18]. Before calculating these indicators, length was converted into height if a child whose age was two years or older. Grossly improbable z-scores were excluded before data analysis (HAZ: <− 6 or > 6; WAZ: <− 6 or > 5; WHZ: <− 5 or > 5).

Bivariate analyses were performed using *t* tests for continuous outcome variables and χ^2^ tests for categorical variables. A multiple logistic regression model was used to assess the influence of the intervention on each of the child health care outcomes (including: early initiation of breastfeeding, exclusive breastfeeding, child feeding index, cook food separately for children, attendance rate at a health service for cold symptoms in two weeks, attendance rate at a health service for diarrhea in two weeks, vitamin A supplementation intake rate, hepatitis B vaccination rate, stunting, underweight and wasting), after adjusting for economic and sociodemographic factors. In the logistic regression, we defined each outcome 0 = no and 1 = yes. Covariates were defined as follows: group 0 = control and 1 = intervention; place of birth 0 = not in township or higher level hospital and 1 = in township or higher level hospital; child’s age, mother’s education, wealth index and size of family were treated as continuous variables.

SPSS Version 13.0 (Statistical Package for Social Science, Inc., Chicago, IL, USA) was used for all analyses. The significance of hypothesis testing was determined as *P* < 0.05 with two-tailed tests.

### Human participants and ethical considerations

The project was approved by the Human Research Ethics Committee of the Xi’an Jiaotong University College of Medicine prior to conducting the research. Written consent was received from the parents of the children who voluntarily participated in the study. All the data collected were kept confidential.

## Results

### Sample characteristics

In the 2001 survey, the information from 2673 (92.8% of the total survey participants) children represented the intervention group and 9031 (97.3%) children for the control. In the 2005 survey, the information from 2693 (93.5%) children represented the intervention group and 8425 (90.8%) children for the control. The following were excluded from the analysis: children or their parents who were unavailable for measurement, children whose birth date was unknown, and children with grossly improbable Z-scores. Table 2 shows the characteristics of the participants before and after the intervention.

**Table 2:** Socio-demographic characteristics of participants in 2001 and 2005

Characteristics	2001				2005			
	Intervention	Control	*T / X* ^2^	*P*-value	Intervention	Control	*T / X* ^2^	*P*-value
	(*n* = 2673)	(*n* = 9031)			(*n* = 2693)	(*n* = 8425)		
Age of child in months,mean *(SD)*	18.6 (9.2)	18.5 (9.9)	0.466	0.641	15.8 (9.5)	17.9 (9.9)	9.675	0.001
Male sex, *n (%)*	1564 (58.5)	5170 (57.2)	1.141	0.285	1579 (58.6)	4858 (57.7)	0.790	0.374
Place of birth, in hospital, *n (%)*	1057(39.5)	4944 (54.7)	190.7	0.0001	2452 (91.1)	6914 (82.1)	124.1	0.0001
Age of mother in years, mean *(SD)*	27.0 (3.9)	26.6 (4.0)	4.567	0.001	27.1 (4.4)	27.2 (4.9)	0.944	0.345
Mother’s education in years, mean *(SD)*	5.8 (3.2)	6.0 (3.2)	2.838	0.004	6.9 (2.9)	6.5 (3.1)	5.919	0.001
Household wealth index, *(SD)*	−0.01 (0.05)	−0.00 (0.06)	7.84	0.001	0.02 (0.03)	0.01 (0.06)	8.322	0.001
Size of family, *n (SD)*	4.3 (1.5)	4.7 (1.6)	11.51	0.001	4.6 (1.6)	4.8 (1.6)	5.646	0.001
Drinking Water Source,, *n (%)*	728 (27.2)	2365 (26.2)	1.164	0.281	1023 (38.0)	3367 (40.0)	3.338	0.068

^1^*P*-values for continuous variables were calculated by Student’s 2-sided t test; *P*-values for categorical variables were calculated using Pearson’s χ^2^ test

### Feeding practices

In 2001, early initiation of breastfeeding occurred in 15.4% of the intervention group and 20.0% of the control group (*p* < 0.001). After the intervention, in 2005, the early initiation of breastfeeding had improved to 51.9% in the intervention group, which was higher than in the control group (41.8%, *p* < 0.001). There was no difference between the two groups in the prevalence of exclusive breastfeeding in 2001 (13.8% vs 14.1%, *p* > 0.05). In 2005, this increased to 24.7% in the intervention group, which was significantly higher than in the control group (14.6%, *p* < 0.001) (Table 3).

**Table 3:** Intervention effects on feed practices, child care, and anthropometric status of children in 2001 and 2005: Univariate analysis

Outcomes (%)	2001				2005				Change	
	Intervention	Control	*X* ^2^	*P*-value^1^	Intervention	Control	*X* ^2^	*P*-value^a^	Intervention	Control
Feed practices										
Early initiation of breastfeeding	15.4	20.0	41.02	0.0001	51.9	41.8	84.52	0.0001	36.5	21.8
Exclusive breastfeeding	13.8	14.1	0.145	0.703	24.7	14.6	147.06	0.0001	10.9	0.5
Child feeding index (score ≥ 6)	5.2	10.0	58.56	0.0001	85.2	80.0	36.00	0.001	80.0	70.0
Child care^a^										
Give separate cooking for children, *> 6 mo*	59.6	55.9	11.50	0.001	73.1	54.4	295.41	0.0001	13.5	−1.5
Attendance rate at a health service for cold symptoms in two weeks^b^	91.4	94.0	22.68	0.001	97.6	95.1	30.71	0.001	6.2	1.1
Attendance rate at a health service for diarrhea in two weeks^c^	81.2	82.5	2.465	0.116	93.2	91.7	6.29	0.01	12	9.2
Vitamin A intake rate	9.2	37.4	767.3	0.0001	89.1	86.4	13.05	0.001	79.9	49
Hepatitis B vaccination rate	82.3	66.2	253.9	0.0001	96.9	97.1	0.247	0.619	14.6	30.9
Anthropometric status										
Stunting	25.4	26.9	2.360	0.124	13.1	18.0	34.96	0.001	−12.3	−8.9
Underweight	12.0	10.5	4.876	0.027	5.6	7.8	14.54	0.001	−6.4	−2.7
Wasting	3.7	3.0	3.32	0.068	3.1	4.1	5.65	0.017	−0.6	1.1

^a^P-values were calculated using Pearson’s χ^2^ test

^b^proportion of attending at a health service for cold symptoms among children who had symptoms of cold in two weeks

^c^proportion of attending at a health service for diarrhea among children who had diarrhea in two weeks

**Fig. 1:**
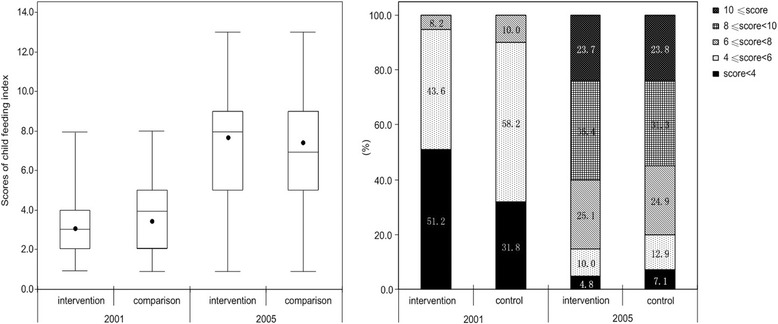
Distribution of scores of child feeding index in 2001 and 2005

The median of child feeding index score varied from 3 to 8 in intervention group, and which varied from 4 to 7 in control group. Before the intervention, the mean child feeding score for the intervention group was lower than that for the control group (3.0 vs 4.0, *p* < 0.001), while after the intervention, the mean score was higher for the intervention group (8.0 vs 7. 0, *p* < 0.001) (Fig. 1).

### Child care

Table 3 shows that more than half of the mothers were cooking separate meals for their children in 2001. After the intervention, more mothers in the intervention than in the control group were cooking separately for their children (73.1% vs 54.4%, *p* < 0.001). The attendance rate at a health service for colds/diarrhea in a two-week period also improved in the intervention group, but the rates of diarrhea cases showed no significant difference in 2005. Vitamin A supplementation intake rates improved significantly, rising to 79.9% in the intervention and 49.0% in the control. The Hepatitis B vaccination rate showed no significant difference between the intervention and control group in 2005 (96.9% vs 97.1%, *p* > 0.05).

### Anthropometric status

There was no difference in stunting or wasting for the two groups in the 2001 (Table 3), although there was a small difference for underweight with the prevalence in the intervention group 1.5% lower than in the control. Following the intervention, all three anthropometric indicators in 2005 in the intervention group were better than in the control, with stunting 4.9% lower (*p* < 0.001), underweight 2.2% lower (*p* < 0.001), and wasting 1.0% lower (*p* < 0.05).

### Evaluation of intervention

The difference between two groups in 2005 was modeled in Table 4. It shows that for child health care behaviors, the intervention group made much more progress than did the control. After adjusting for the socio-demographic characteristics, we found that the intervention was associated significantly with early initiation of breastfeeding, exclusive breastfeeding, child feeding index (score ≥ 6), giving separate cooking for children, attendance at a health service for cold symptoms in two weeks, and vitamin A supplementation intake. The prevalence of stunting was reduced by 3.4% more in the intervention group than in the control (Odds ratio: 0.82, 95%CI: 0.72 to 0.94), and wasting was reduced by 1.7% of more in intervention than in control (Odds ratio: 0.75, 95%CI: 0.59 to 0.97). However, for the attendance at a health service for diarrhea in two weeks and underweight, no significant intervention effects were observed (*p* > 0.05).

**Table 4:** Intervention effects on feed practices, child care, andanthropometric status of children in 2005: Multiple logistic

Outcomes	Odds ratio (95% CI)	*P*-value
Feed practices		
Early initiation of breastfeeding	1.30 (1.19,1.42)	< 0.001
Exclusive breastfeeding	2.32 (1.72, 3.13)	< 0.001
Child feeding index (score ≥ 6)	1.12 (1.01, 1.25)	< 0.05
Child care		
Give separate cooking for children	1.79 (1.62, 1.97)	< 0.001
Attendance at a health service for cold symptoms in two weeks	2.16 (1.07, 4.37)	< 0.05
Attendance at a health service for diarrhea in two weeks	1.12 (0.51, 2.48)	NS
Vitamin A supplementation intake	1.16 (1.02, 1.31)	< 0.05
Hepatitis B vaccination	1.20 (0.78, 1.84)	NS
Anthropometric status		
Stunting	0.85 (0.74, 0.97)	< 0.05
Underweight	0.85 (0.71, 1.03)	NS
Wasting	0.75 (0.58, 0.97)	< 0.05

The multiple logistic regression model was used to assess intervention effect of the cross sectional results in 2005 on each outcome after adjusting for child’s age, mother’s education, wealth index, size of family and child’s birth place

## Discussion

This four-year child health care intervention was aimed to promote child growth and reduce the incidence of malnutrition of infants and young children by encouraging recommended feeding and dietary practices, as well as to improve the rate of Hepatitis B vaccination and the vitamin A supplementation intake rate in rural China.

For child growth the intervention seemed successful in reducing the prevalence of stunting. The intervention group saw the risk of stunting lowered by 12.3%. This result could be explained by the following reasons. First, breastfeeding was reported to have a stronger effect on linear growth than on weight gain during late infancy and preschool years [19]; Second, primary disease prevention education was part of the intervention, and may have resulted in improved hygiene and sanitation, which have also been reported to improve linear growth [20]; and third, the increased intake of complementary foods such as milk-based gruels or cereal-pulse mixes may represent improved nutrient quality intake [21]. The effect of intervention on reducing the prevalence of stunting should be interpreted with the fact that rapid economic growth occurred in China from 2001 to 2005 with increase over 8.0% per year.

In the community-based health education program, the messages need to be well disseminated so as to reach the target population. This study demonstrates the value of using the existing primary health care management channels of “county-township-village” for message delivery, wide dissemination of messages related to optimal exclusive breastfeeding, and instruction on complementary feeding practices and other child health care practices. This was a government-led project, so the assessment results of project progress reflected on the work performance of government health officials. As such, strong political support was consistently provided throughout the project. Another feature of this intervention was that the government was responsible for training and supervising those involved in carrying out the project, the village doctors in particular, and for hiring MCH complementary foods specialists at the county level to supervise and support the village educators until the feeding practices had improved more widely [22]. This study not only reached large numbers of the target audience but also reached them frequently enough to stimulate lasting behavioral changes. For an organization with limited funding, it can be very difficult to devote a large amount of manpower and a long period of time to complete such a project as this one without support from different levels of government.

The majority of child feeding practice studies have focused their evaluations on single behaviors, and it may be difficult to summarize a series of interrelated behaviors into one or a few variables that accurately reflect these practices. Based on a study by Ruel [15], we used the child feeding index to assess child feeding behaviors. Using this indicator, we were able to conduct a quantitative evaluation of the intervention effect on child feeding behaviors. Even though a study conducted in rural Senegal reported that a young child feeding index was not associated with either height-for-age or height velocity [23]. Another Chinese study found significant correlations between the feeding index and height-for-age or weight-for-age [16].

In this study, although early initiation of breastfeeding and exclusive breastfeeding among the intervention group showed more improvement than among the control, the results were still not as significant as we had expected. In 2005, the intervention group’s prevalence of early initiation of breastfeeding was about 50% but the rate of exclusive breastfeeding was no more than 25%. The same phenomenon was observed in previous studies in rural China [22] but there was a very strong secular trend to having delivery in hospital among both the intervention and control groups. Early initiation of breastfeeding is easier in hospital-based deliveries.

Traditional beliefs regarding child feeding practices may be the main reason for the relatively low rates of breastfeeding. In the study areas, we found that mothers used to be restricted to bed rest for an extended period of time after giving birth, believing that breastfeeding should not begin immediately after delivery. As a result, many women feed newborn infants with only water or sugar water. Furthermore, in rural areas, mothers involvement in farm work limited them to commit to exclusive breastfeeding.

After adjusting for socio-demographic characteristics, we found that the intervention significantly increased the attendance rate at a health service for cold symptoms in two weeks; but for attendance rate at a health service for diarrhea, the intervention results were not satisfactory. There are some possible explanations for this. Through education, parents can more easily recognise the symptoms of a cold, and take a child immediately for treatment. Although most cases of diarrhea in children can be discovered in a timely manner, due to economic factors among rural residents, non-severe diarrhea is often not considered a condition requiring treatment.

This study had some limitations. First, the intervention and control groups were not randomly allocated. As a result, the observed treatment differences may be subject to unobserved confounding factors. Second, during the intervention, although the household wealth index was used, we could not completely rule out the confounding factor of concurrent socioeconomic development that would also improve child health care conduct within the control group. Third, another confounding factor arose from our finding was that the hepatitis B vaccination rate of the control group was higher after the intervention. This is because during the implementation of the intervention, the Chinese government carried out a hepatitis B vaccination program free of charge nationwide. Therefore, it is likely that the health education initiative was not responsible for any significant effect on the vaccination rate. Fourthly, some findings were based on participants’ subjective response. They were not as objective as growth outcomes because the intervention emphasizes them. Fortunately, outcomes based on participants’ response showed similar pattern as growth outcomes. Two types of outcomes could serve the purpose to evaluate the effect of health education.

## Conclusion

Based on the results from this study, we concluded that this health care education intervention embed in government had the potential to be successfully promoted in rural western China. Community-based intervention on child feeding, child health care and child growth can effectively promote the growth and development of children.

## Additional files

Additional file 1: The questionnaire. (DOC 313 kb)

Additional file 2: Interview guide. (DOC 212 kb)

## Abbreviations

CI: Confidence interval
HAZ: Height-for-age
RPHC: Rural primary health care project
UNICEF: United nations international children’s emergency fund
WAZ: Weight-for-age
WHO: World health organization
WHZ: Weight-for-height
