# Supplementary material for: Community health education improves child health care in Rural Western China
Source: BMC Pediatr. 2018 Apr 10;18:132. doi: 10.1186/s12887-018-1084-0 (PMC5891978; doi:10.1186/s12887-018-1084-0)
Supplement: Supplementary file 1 — The questionnaire. (DOC 313 kb) [file 12887_2018_1084_MOESM1_ESM.doc]

**Household questionnaire**

(Enrolling investigate the mother with child under 3 years old)

| Village:  Code of the mother:  Name of the mother: post code | | |
| --- | --- | --- |
| **Variables** | Contents | **Code** |
| BD | Date interview (solar calendar): y m d |  |
| B4 | The child’s birthday (solar calendar) y m d  (Lunar calendar) y m d |  |
| B1 | How many members are there in your family? |  |
| B2 | Your race: ①The Han nationality ②others |  |
| *B57* | *What is your birth date:（solar calendar） y m d*  *（lunar calendar ） y m d* |  |
| *B58* | *How many years of education have you received years* |  |
| *B59* | *How many years of education have your husband received years* |  |
| B3 | Have you TV set in your family? ①Color②monochrome③No |  |
| B54 | Who informed you that we would interview you?  ①village doctor②head of the village or the cadre③MCH cadre④others |  |
| B55 | When did you being informed the time of interview? ①today②yesterday③the day before yesterday④three days ago⑤more earlier( month day) |  |
| B56 | Did there anybody suggest you how to answer the questions? ①Yes ②No |  |
| B52-1 | Did any of your family members saw the doctor in the village clinic from Jan. to Jun. this year? ①Yes ②No(to B43-1) |  |
| B52-2 | If “Yes”, did he or her accepted transfusion (including mainline)? ①Yes ②No |  |
| B43-1 | Did you receive antenatal care when pregnancy? ①Yes ②No(to B44-1) |  |
| *B60-1*  *B60-2*  *B60-3*  *B60-4* | *When you have examine before bearing, the track record is saved by yourself?* ① *yes* ② *no* ③ *unknown*  *If yes, do you take the same card of health care every time?*  *① yes ② no ③ unknown*  *Do you think the card give you useful information?*  *① yes ② no ③ unknown*  *Did the sanitation personnel who delivered a child for you has let you see the card? ① yes ② no ③ unknown* |  |
| B43-2 | Where did you receive the antenatal care? ①At home②Village clinic③Township hospital④MCH hospital of county level and above⑤general hospital on county level and above⑥others |  |
| B44-1 | Did anyone give you guidance on health care during pregnancy (such as antenatal care or breast feeding)? ①Yes ②No |  |
| B44-2 | Did anybody suggest you to give birth in hospital? ①Yes ②No(to B45-1) |  |
| B44-3 | If “Yes”, who gave the suggestion? (multiple selection) ①village doctor or health care worker②women cadre③Family Planning worker④family member, relatives and neighbor ⑤others |  |
| *B61-1*  *B61-2*  *B61-3*  *B61-4* | Did you be cared on bite and sup by your family before produce?  ① *yes* ② *no* ③ *unknown*  Do your quantity of bite and sup increase before produce?  ① *yes* ② *no* ③ *unknown*  Do your qualty of bite and sup increase before produce?  ① *yes* ② *no* ③ *unknown*  Were your labor before produce including housework little than peacetime? ① *yes* ② *no* ③ *unknown* |  |
| B45-1 | Where did the child born? ①At home②Village clinic③Township hospital④MCH hospital of county level and above⑤general hospital on county level and above⑥others |  |
| B45-2 | Was that delivery smoothly? ①Yes②No③Not known |  |
| B46 | If you delivery at home, who is the birth attendance? ①family member, relatives and neighbor ②TBA ③village doctor④Doctor of Township hospital |  |
| B48-1 | The reason that you didn’t delivery in hospital (multiple selection)   1. Problem of charge②Problem of traffic③others   (If you delivery in hospital please fill in “999”) |  |
| B47-1 | If you delivery in the township hospital and above please answer the questions: |  |
|  | Who made the decision that you should delivery in hospital? ①Husband②mother-in-law③Yourself④village doctor or TBA⑤Others |  |
| B47-4 | You stay in the hospital for days |  |
| B47-3 | The total charge in the hospital is Yuan |  |
| B47-2 | The total expenditure (including traffic, accommodation and medical treatment) is Yuan  Did you have any special situation? Yes |  |
| B48-3 | If it is free charge for hospital delivery, would you like to go to the hospital? ①Yes ②No |  |
| B49-1 | How did you evaluate the attitudes of the medical staffs when you delivery in the hospital? ①Very kind②Kind③Not kind |  |
| B49-2 | How did you evaluate the live during you delivery in the hospital (such as food, company and cloth washing etc.)? ①Very convenient②Convenient③Not convenient |  |
| B5-1 | Have this child ever been vaccination or taken TOPV? ①Yes②No③Not known |  |
| B5-2  *B5-3* | Have any record of the vaccination? ①Yes②No③Not known  *Have this child ever been vaccination below: (multi choice) ①.no ②. BCG ③. TOPV ⑤.Measles ⑥. HBV* |  |
| B7-1 | Did this child accept the HBV vaccination? ①Yes②No③Not known |  |
| B7-2 | If accepted, the charge of the complete process is Yuan.  (If you don’t know exactly, please fill in 9.99) |  |
| B8 | Did the village doctor weigh in the child this year? ①Yes②No (to B13) |  |
| B9 | If “Yes”, did you know the result? ①Yes②No(to B13) |  |
| B10 | If “Yes”, is the weigh reach to the standard? ①Yes②No③Not known(to B13) |  |
| B11 | If ”No”, what did the doctor suggest to take more? (no hint and multiple selection) ①egg②vegetable③meat④medicine(such as calcium tablet and vitamins)⑤something nutritious⑥say nothing |  |
| *B62-1*  *B62-2* | *Do you give attention to the increase of your children’s avoirdupois?*  *①No ②occasional ③Often*  *If a child’s weight do not increase in two months, you think the main reasons (multi choice) (may explain) ① adding supplement foods not on schedule ② not enough quantity ③ inferior quality ④ Illness ⑤particular on food* |  |
| B13 | When did the baby supplement egg? Months  If you didn’t supplement fill in 99, supplement while born fill in 00 |  |
| B14 | If you have supplement egg, how did you do it? ①Every day②2-3times a week③once a week④2-3 times a month⑤once a month⑥less than once a month |  |
| B 15 | When did the baby supplement meat and liver (pork, beef, mutton, fish and chicken) Months  If you didn’t supplement fill in 99, supplement while born fill in 00 |  |
| B16 | If you have supplement, how did you do it? ①Every day②2-3times a week③once a week④2-3 times a month⑤once a month⑥less than once a month |  |
| B53-1 | Did any of your family members went to the village clinic in the latest 30 days? ①Yes②No(to B17) |  |
| B53-2 | If “Yes”, the name of the patient is  Try to find the prescriptions of this Person,  ①Yes, it is found②No, it couldn’t be found③ We don’t try it, the reason is |  |
| B17 | Did your child catch a cold? ①Yes②No(to B28) |  |
| B18 | Have your child caught a cold within 2 weeks? ①Yes②No |  |
| B19 | Did the child receive treatment when recently caught a cold? ①Yes②No(to B28) |  |
| B20 | If “Yes”, where did the child get the treatment? ①To buy the medicine yourself(to B28)②village clinic(non private)③private practitioner④Township hospital⑤hospital on county level⑥others |  |
| B21 | Did the child receive the injections? ①Yes②No |  |
| B22 | Did the child receive the transfusion (mainline)? ①Yes②No |  |
| B23 | Did the doctor prescribe? ①Yes②No |  |
| B25 | You pay for the latest cold is Yuan |  |
| B26-1 | Did you pay for the registration? ①Yes②No③Not known(to B27) |  |
| B26-2 | If “Yes”, how much is it? Yuan. (Not know fill in 9.9) |  |
| B27 | Was it a cold with diarrhea? ①Yes②No |  |
| *B63* | *Which symptom do you think is belong to children pneumonia and should call on Dr at once: ①breathe rapidly ② high fever ③ cough fiercely ④ other* |  |
| B24 | Try to find the prescription if caught cold and get treatment within 2 weeks. ①Yes, it is found②No, it couldn’t be found③ We don’t try it, the reason is |  |
| B28 | Did child get diarrhea? ①Yes②No(to B39)  Have your child got diarrhea within 2 weeks? ①Yes②No |  |
| B29 | Did the child receive the treatment when diarrhea recently? ①Yes②No(to B39) |  |
| B30 | If “Yes”, where did the child get the treatment? ①To buy the medicine yourself(to B28)②village clinic(non private)③private practitioner④Township hospital⑤hospital on county level⑥others |  |
| B31 | Have the child take ORS (including self-made)? ①Yes②No |  |
| B32 | Have the child take injection? ①Yes②No |  |
| B33 | Have the child take transfusion (including mainline)? ①Yes②No |  |
| B34 | Did the doctor prescribe? ①Yes②No③Not known |  |
| B35 | The total cost of the latest diarrhea is Yuan |  |
| B36 | Did you pay for the registration? ①Yes②No③Not known(to B27) |  |
| B37 | If “Yes”, how much is it? Yuan. (Not know fill in 9.9) |  |
| B38 | Try to find the prescription if diarrhea and get treatment within 2 weeks. ①Yes, it is found②No, it couldn’t be found③ We don’t try it, the reason is |  |
| *B64* | *What do you prefer when your children have a cold or diarrhea ?*  *①take medicine ②injection③transfusion④follow the doctor’s advice* |  |
| B39 | Where do you prefer to see the doctor when your child ill? ①private practitioner ②village clinic③Township hospital④hospital on county level and above⑤MCH hospital on county level and above⑥others |  |
| B40 | Why did you select this? ①kind attitude ②cheap③high quality of the doctor④nearby⑤convenient⑥others |  |
| *B65-1*  *B65-2*  *B65-3*  *B65-4* | *Do you think your information of feeding children is enough?*   1. *Yes②No*   *If yes, your approach ①TV ②books and newspaper ③ doctor ④member of family and section ⑤other*  *Do you learn the information by yourself or by someone else?*   1. *Initiative* ② *passive*   *If no, you prefer to obtain the information from?*  *①TV ②books and newspaper ③ doctor ④member of family and section ⑤other* |  |
| *B66-1*  *B66-2*  *B66-3* | *Do you know how old a child can creep? ①<6 month ②6-9 month ③9-12 month ④12-18 month ⑤18-24 month ⑥unknow*n  *If a child can not creep when he is one year old, you think the main reason ① develop slowly ②badly nutrition ③ Illness ④ mental retardation ⑤unknown*  *If this case happened, you think: ①individual difference and never mind ② have a question but can wait a while ③severity and see a doctor immediately ④ unknown* |  |
| *B67-1*  *B67-2* | *Do you play with your children at home? ① never ② occasional ③sometimes ④ often ⑤ always*  *Do you think talking and playing with children under one year old is important? ① no ② common ③ important ④very important* |  |
| B41 | What do you prefer when you are ill? ①take medicine ②injection③transfusion④follow the doctor’s advice⑤others |  |
| *B68* | *Do you know the system of cooperative medical treatment*  *① Yes②No* |  |
| *B69-1*  *B69-2*  *B69-3* | *Did your family take part in the new pattern rural cooperative medical treatment? ①Yes ②No*  *If yes, the expenses of cooperative medical treatment is _____ ￥ each person*  *If no, the reason of nonparticipation*  *①the family are healthy②economic embarrassment ③wait-and-see*  *④didn’t know* |  |
| *B70* | *the payment of cooperative medical treatment is*  *①voluntary ②voluntary after agitprop*  *③under pressure ④forced ⑤others* |  |
| *B71-1*  *B71-2*  *B71-3* | *the proportion to apply for the reimbursement of the expenses of cooperate medical treatment in this country*  *in the township clinic the proportion is*  *in the township hospitalization proportion is*  *in the country hospital the proportion is* |  |
| *B72* | *the form of reimbursement of cooperative medical treatment*  *① reimbursement immediately ② reimbursement at any moment*  *③ reimbursement at fixed time ④don’t know* |  |
| *B73* | *the times of reimbursement of fee-for-service last year is*  *①0 ②1 ③ 2 or 2 more* |  |
| *B74* | *your attitude to the cooperative medical treatment*  *①uphold greatly, solve the problem that peasant can’t afford the fee-for-service*  *②uphold relatively, solve part of the problem that peasant can’t afford the fee-for-service*  *③ hold my attitude ④ unclear* |  |
| *B75* | *Is there anyone given you the publicizing and instruction of cooperative medical treatment ①yes ② no* |  |
| *B76* | *whether did you sign the compact of participation*  *①yes ②no* |  |
| *B77* | *Are you satisfied with the attitude and quality of the service since the cooperative medical treatment*  *①satisfy ②satisfy relatively ③dissatisfied* |  |
| *B78* | *how about your intent to join in the cooperative medical treatment next year*  *①continue ②nonparticipation ③haven’t decision ④others* |  |
| *B79* | *if select ② ,the reason of nonparticipation (multi choice)*   1. *economic difficulty ②small proportion of reimbursement*   *③lack of supervision and belief to the management and the use of outlay ④the procedure of reimbursement is inconvenient*  *⑤sane or seldom fall ill ⑥others* |  |

| Measurement | | |
| --- | --- | --- |
| B80 | The impression of children   1. Clean ②ordinary ③No Clean | — |
| B81  B82  B83  B84  B85 | Height of mother__ __ __ .__cm  Weight of mother and children__ __ .__kg  Weight of mother __ __ .__kg  Weight of children __ __ .__kg  Height of children __ __ __.__cm | __ __ __ .__  __ __ .__  __ __ .__  __ __ .__  __ __ __ .__ |
| B86  B87 | HB of mother __ __.__g/dL  HB of children __ __.__g/dL | __ __ .__  __ __ .__ |
| B88 | The cooperating attitude of the interviewee①Very good②Good③Not good④Bad | __ |
| Auditing | Date of the first auditing signature  Date of the second auditing signature  Date of the third auditing signature | |

Questionnaire for village hospital

(Answered by the dean or PMC workers)

| Village  Code  Name of the dean Post code Tel | | | |
| --- | --- | --- | --- |
| **Variables** | Contents | | **Code** |
| CT | Date interview (solar calendar):2005 Y D | | 2005 / / |
| The following questions are concerning about the basic situation of the village, which should be answered by the staff from local government. | | | |
| C1 | The distance from the township to the county seat  is kilometers |  | |
| C2 | The population at the end of 2003 in the township is |  | |
| C3 | The income per capita in 2003 is Yuan |  | |
| C4 | administrative townships included. |  | |
| C5 | The number of living neonates in 2003 is |  | |
| C6 | The IMMR in 2003 is |  | |
| The following focusing on the situation of the village hospital. | | | |
| C7 | The annual net earning in 2003 is Yuan | |  |
| C8-1 | The total expenditure of medicine purchase in 2003 is  Yuan | |  |
| C8-2 | Among this, Yuan is for the village clinic | |  |
| C9 | What the township hospital turned in is Yuan in 2003 | |  |
| C10 | The earning turned in is in the name of  ①percentage taken ②management charge③pay the taxes④others(listed) | |  |
| C11-1 | Which is the department in charge of expense?  ①county level health bureau ②local government③being private, stocking or contracting | |  |
| C11-2 | Which is the department in charge of personnel?  ①county level health bureau ②local government③being private, stocking or contracting | |  |
| C12 | The wage payable of the township hospital is Yuan from Jan. to Jun. in 2004 | |  |
| C13 | The appropriate wages from the local government  are Yuan | |  |
| C14 | Actually, the output for salaries in the township hospital  is Yuan from Jan. to Jun. in 2004 | |  |
| C15 | Could the PHC workers get the full-wage? ①Yes②No | |  |
| C16-1 | Is the township integrate managed? ①Yes②No | |  |
| C16-2 | If ”Yes”, when did it begin? (year) (month) | |  |
| C16-3 | What is the content of it?  ①unified medicine purchase ②unified medical price③unified personnel arrangement④unified wages paid⑤unified qualification identified | |  |
| C34-1 | Do the hospital perform birth attendance? ①Yes②No | |  |
| C34-2 | If ”Yes”, do you have the special delivery ward (The inquirer should site investigate)? ①Yes②No | |  |
| C34-3 | The number of obstetrical staffs is | |  |
| C34-4 | The number of obstetrical staffs with technical secondary school educational level is | |  |
| C34-5 | The number of obstetrical staffs with one experience  is | |  |
| C17-1 | The number of hospital delivery women in 2004 is | |  |
| C17-2 | Among these, the number in township hospital is | |  |
| C17-3 | The average expenditure for a normal hospital delivery is  in township hospital. | |  |
| C18-1 | The outpatient quantity of the township hospital in 2004  is person-time. | |  |
| C18-2 | There are beds inside. | |  |
| C18-3 | The number of inpatient in 2003 is person -time | |  |
| C19-1 | Was there inpatient record in May 2004 in the hospital? ①Yes②No | |  |
| C19-2 | If ”Yes”, the number of the inpatient is | |  |
| C19-3 | Was there outpatient record in May 2004 in the hospital? ①Yes②No | |  |
| C19-4 | If ”Yes”, the number of the outpatient is | |  |
| C19-5 | There were prescriptions in May 2004 totally. | |  |
| C19-6 | Were they the unified project prescriptions? ①Yes②No | |  |
| C20-1 | Do you charge the registration? ①Yes②No | |  |
| C20-2 | If ”Yes”, it is Yuan once for each person | |  |
| C21-1 | Which kind of syringe used for the injection?  ①One-off syringe ②Non one-off syringe③Both | |  |
| Next we would like learning about the purchase and sale of the medicine. | | | |
| C22-1 | Where did you buy the medicine in May this year?  ①Medical company of county level ②Provincial medical company③Private medical seller | |  |
| C22-2 | Who is responsible for the medicine purchase in your hospital?  ①County level②Township level | |  |
| C22-3 | Who is responsible for the medicine purchase in village clinic?  ①County level②Village level③Village level | |  |
| C23-1  C23-2 | The purchase price for one 800 thousand unit penicillin  is Yuan  And sale price of it is Yuan. | |  |
| C24-1  C24-2 | The purchase price for one one-off 5ml syringe is Yuan  And sale price of it is Yuan.  If you don’t have either of them, please fill in 9.99 | |  |
| C25 | Did the retail price in May 2004 decided unified by the county level? ①Yes②No | |  |
| C26 | Was there a public declaration of the medicine retail price? ①Yes②No | |  |
| The following is about the elementary medical equipment. | | | |
| C27 | The batheroom scale for children ①Yes②No | |  |
| C28 | ORS ①Yes②No | |  |
| C29-1 | Could the pregnant women with acute compliance use the vehicle in emergency (such as transfer the pregnant/lying-in women to hospital)? ①Yes②No | |  |
| C29-2 | If “Yes”, please tell me the telephone number of the first aid (Fill in 99 if you don’t know and with 00 if you have none) | |  |
| C29-3 | Do you charge for the vehicle? ①Yes②No | |  |
| C29-4 | If “Yes”, the charge for one time is Yuan | |  |
| C29-5 | Or the charge for one kilometer is Yuan | |  |
| C29-6 | Or the charge for one hour is Yuan | |  |
| C30-1 | The subsidy from project you village received in 2003  is Yuan | |  |
| C30-2 | Among this, Yuan is for the village level | |  |
| C31 | The county level leaders monitored and evaluated the project work in your hospital for times from Jan. to Jun. in 2004. | |  |
| C32-1 | Do you village take on the HBV vaccination? ①Yes②No | |  |
| C32-2 | If “Yes”, the total charge of the complete process  is Yuan | |  |
| *C33* | *Did we bring cooperative medical treatment into cadre’s annual examining responsibility book in 2003?*  *①Yes ②No* | |  |
| *C34* | *How many people joined cooperative medical treatment last year?* | |  |
| *C35-1*  *C35-2*  *C35-3* | *Regard village as the unit*  *The joining rate of cooperative medical treatment in 2001 is ％*  *The joining rate of cooperative medical treatment in 2002 is ％*  *The joining rate of cooperative medical treatment in 2003 is ％* | | *％*  *％*  *％* |
| *C36-1*  *C36-2* | *The surplus of cooperative medical treatment outlay in 2003 is*  *The overspending of cooperative medical treatment outlay in 2003 is* | |  |
| *C37-1*  *C37-2*  *C37-3* | *The first three disease diagnosed in 2003 are*  *1、*  *2、*  *3、* | |  |
| *C38-1*  *C38-2*  *C38-3* | *The first three disease applied for reimbursement in cooperative medical treatment in 2003 are:*  *1、*  *2、*  *3、* | |  |
| *C39* | *Did we established management system of fee controlling and service quality ensuring?*  *①Yes ②No*  *Specific measure* | |  |
| *C40* | *What is the format of applying for reimbursement in cooperative medical treatment in 2003?*  *① derate on the spot ②apply for reimbursement at*  *any time ③ apply for reimbursement at a certain time* | |  |
| *C41-1*  *C41-2* | *The proportion of applying for reimbursement in countryside hospital:*  *outpatient*  *inpatient* | |  |
| *C42-1*  *C42-2*  *C42-3* | *The content of cooperative medical treatment*  *Medicine and drug （number of villages）*  *Medicine but not drug （number of villages）*  *Drug but not medicine （number of villages）* | |  |
| *C43* | *The percentage of people who got medical compensation in all the people joined the insurance ％* | |  |
| C33 | The cooperating attitude of the interviewee is  ①Very good ②Good③Not good④Bad | |  |
| Thank you for the cooperation!  Remark and the hindrance  Date interview signature of the inquirer  Date auditing signature  Date auditing signature | | | |

**The records of the 30 prescriptions from the township** hospital

| No. | **Total amount of medicine taken** | **Amount of antibiotic** | **Amount of hormone** | **Amount of muscle injection** | **Amount of mainline** | **Price** |
| --- | --- | --- | --- | --- | --- | --- |
| 1 |  |  |  |  |  |  |
| 2 |  |  |  |  |  |  |
| 3 |  |  |  |  |  |  |
| 4 |  |  |  |  |  |  |
| 5 |  |  |  |  |  |  |
| 6 |  |  |  |  |  |  |
| 7 |  |  |  |  |  |  |
| 8 |  |  |  |  |  |  |
| 9 |  |  |  |  |  |  |
| 10 |  |  |  |  |  |  |
| 11 |  |  |  |  |  |  |
| 12 |  |  |  |  |  |  |
| 13 |  |  |  |  |  |  |
| 14 |  |  |  |  |  |  |
| 15 |  |  |  |  |  |  |
| 16 |  |  |  |  |  |  |
| 17 |  |  |  |  |  |  |
| 18 |  |  |  |  |  |  |
| 19 |  |  |  |  |  |  |
| 20 |  |  |  |  |  |  |
| 21 |  |  |  |  |  |  |
| 22 |  |  |  |  |  |  |
| 23 |  |  |  |  |  |  |
| 24 |  |  |  |  |  |  |
| 25 |  |  |  |  |  |  |
| 26 |  |  |  |  |  |  |
| 27 |  |  |  |  |  |  |
| 28 |  |  |  |  |  |  |
| 29 |  |  |  |  |  |  |
| 30 |  |  |  |  |  |  |

Neither the glucose as well as the brine nor the syringe as well as injection water and the transfusion included in the drugs when take transfusion.

The meidcine include the glucose and the brine when the patients only take these.

**The questionnaire for the county level**

(Filled by the county level health bureau or units concerned )

| County  Code  (Please fill in the data of your county in 2003 in the middle line, thank you!) | | |
| --- | --- | --- |
| **Variables** | Contents | **Code** |
| D1 | The total number of the administrative townships in the county  is |  |
| D2 | The total number of the administrative villages in the county  is |  |
| D3 | The population of the county is |  |
| D4 | The poverty population in the county is |  |
| D5 | The annual income per capita of the agriculture population in the county is Yuan. |  |
| D6 | The number of children under 3 years old in the county is |  |
| D7 | The number of children under 5 years old in the county is |  |
| *D35-1*  *D35-2*  *D35-3*  *D35-4*  *D35-5* | *the number of the women that who will procreate in the county is*  *the number of the women that whose age is less than 20 is*  *the number of the women that whose age is between 20 to 30 is*  *the number of the women that whose age is between 30 to 40 is*  *the number of the women that whose age is more than 40 is* |  |
| D8 | The number of living neonates in the county is |  |
| D9 | The number of hospital delivery in the county is |  |
| D10 | The number of maternal death in the county is |  |
| D11 | The number of infant death in the county is |  |
| D12 | The number of children death under 5 years old is |  |
| D13-1 | The rate of HBV vaccination in the county is % |  |
| D13-2 | The charge of a complete HBV vaccination is Yuan |  |
| The management of the project and the medical care providing in the county. | | |
| D14-1 | The situation of the finance and personnel management of the township hospital all over the county is (multiple selection)  ①running and management by township level ②runnning and management by county level③others |  |
| D14-2 | The number of township hospitals managed by township level is |  |
| D14-3 | The number of township hospitals managed by county level is |  |
| D14-4 | The number of township hospitals managed by others  is (stocking of contracting). |  |
| D15 | The total outlay received for project management in 2003(including routine meetings held, monitor and evaluation and baseline survey) is Yuan. (The inquirer should check warrant of the appropriate finance) |  |
| D16-1 | The proportion of the personnel wages in township hospital provided by the county government is % |  |
| D16-2 | From the least % to the most %  (the up line refers to the persons, the below refers to the money ) |  |
| D17-1 | The average cost of normal delivery (exclude lateral episiotomy) in the county level hospital is Yuan |  |
| D17-2 | The average cost of caesarean section in the county level hospital is Yuan |  |
| D18-1 | The average cost of normal delivery in MCH hospital  is Yuan |  |
| D18-2 | The average cost of caesarean section in MCH hospital  is Yuan |  |
| D19-1 | There were village clinics (non-private) in the county in 2003 all together. |  |
| D19-2 | The total number of the village doctors is |  |
| D19-3 | The number of village doctors who received the specialty evaluations is . |  |
| D19-4 | Persons was (were) eliminated. |  |
| D19-5 | Did the admittance system of the village doctors put into practice all over the county? ①Yes②No |  |
| D19-6 | The fixed number of year for the practitioners license reexamination is years. |  |
| D20-1 | There are private clinics (exclude village doctors) in the county. |  |
| D20-2 | Of these have the practitioners licenses |  |
| D20-3 | The charge of examination and approve for the private license is Yuan for each. |  |
| D21-1 | Do you have unified essential medicine list on the village level in the county? ①Yes②No |  |
| D21-2 | If “Yes”, there are kinds of western medicine. |  |
| D22-1 | There are townships carried out the integrate management in the county. |  |
| D22-2 | Was the medicine purchased unified? ①Yes②No |  |
| D22-3 | If “Yes”, is the unified organization and purchase carried out only on the township level and above? ①Yes②No |  |
| D22-4 | If “Yes”, which is responsible for the medicine purchase?  ①county health bureau ②other units on county level③township health bureau④other provincial units |  |
| D23-1 | Did village doctors receive the subsidy from county government since the project carried out? (not include the subsidy of project itself) ①Yes②No |  |
| D23-2 | If “Yes”, the subsidy in 2002 is Yuan per month. |  |
| D23-3 | The subsidy in 2003 is Yuan per month. |  |
| D24-1 | There are township hospitals in the county totally. |  |
| D24-2 | Among these, have the midwifery capabilities(including staffs and equipments). |  |
| *D25* | *Have the cooperate medical treatment been brined to the assess book of cadre every year during 2003 ?*  *①yes ② no* |  |
| *D26* | *How many people joined the cooperate medical treatment last year?* |  |
| *D27-1*  *D27-2 D27-3* | *unit is village*  *the joint rate of the cooperate medical treatment in 2001*  *the joint rate of the cooperate medical treatment in 2002*  *the joint rate of the cooperate medical treatment in 2003* | *％*  *％*  *％* |
| *D28* | *the form of reimbursement of cooperative medical treatment is*  *①derate immediately ②reimbursement at any moment*  *③reimbursement at fixed time* |  |
| *D29-1 D29-2* | *the surplus outlay of cooperative medical treatment during 2003*  *the overspend outlay of cooperative medical treatment during 2003* |  |
| *D30-1*  *D30-2 D30-3* | *the first three diseases which were cured in 2003*  *1、*  *2、*  *3、* |  |
| *D31-1*  *D31-2 D31-3* | *the first three diseases which were applied*  *for reimbursement in 2003*  *1、*  *2、*  *3、* |  |
| *D32-1*  *D32-2* | *in the county clinic, the proportion in 2003 is*  *in the county clinic, the proportion is*  *in the country hospital the proportion is* |  |
| *D33* | *Did you establish the supervise system for controlling charge and assuring serving quality in 2003?*   1. *yes ②no*   *Particular measures* |  |
| *D34* | *How many times did you publicize the account of cooperative medical treatment to the villagers last year?*  *① zero ② one ③ two ④ exceed two* |  |
| Name of the inquirer: Date interview: 2005/ / | | |

Signature of the assessor Date 2005/ /
