# Supplementary material for: Community health education improves child health care in Rural Western China
Source: BMC Pediatr. 2018 Apr 10;18:132. doi: 10.1186/s12887-018-1084-0 (PMC5891978; doi:10.1186/s12887-018-1084-0)
Supplement: Supplementary file 2 — Interview guide. (DOC 212 kb) [file 12887_2018_1084_MOESM2_ESM.doc]

**Ministry of Health -UNICEF**

The Rural Primary Health Care Project(**2001-2005)**

Investigation Guide

**
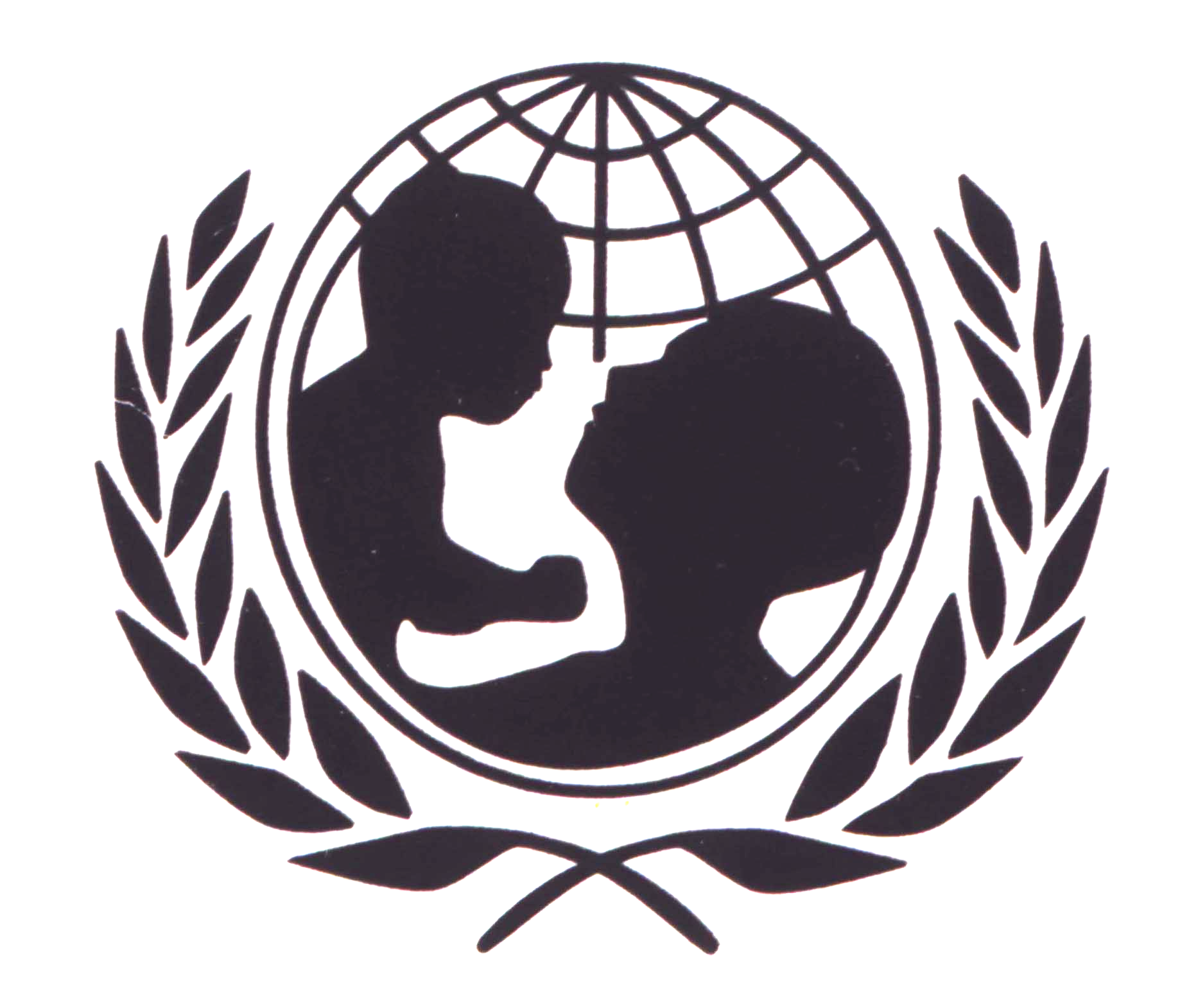
**

**Xi’an Jiaotong University College of Medicine**

**2005.6**

**Objective**

1. To evaluate the effect of community-based intervention project on child feeding, child health care and child growth。

2. To evaluate and monitor the status and implementation of drug use,mother safety and caring for children in rural primary health care project counties.

3. To understand the prevalence of anemia among women and children in project counties and the growth and development status of children and their influencing factors so as to conduct targeted interventions。

**Investigation Area and Population**

34 counties from Guizhou,Gansu,Qinghai,Ningxia, Jiangxi, Guangxi,Sichuan,Inner Mongolia, Xinjiang,Chongqing Province in China.

9 counties were subjected to the intervention and 25 counties were used as controls.In each county, five townships were selected with probability proportional to total population (from the countries’ demographic data). In each selected township, four villages were selected randomly, also with probability proportional to population size. Finally in each village 16 children less than three years old were randomly selected from all the children aged <3 years in this village.

**Questionnaire and Contents**

The questionnaire has been set by UNICEF and the Nitional Center for Women and Children’s Health of China CDC. According to the purpose and content of the survey, there are four kinds of questionnaires in this survey, including the general survey of the county, the township hospital, the village clinics and the family questionnaire.

**Staff selection and training of investigators**

A total of 21 teams surveyed in this investigation. Each team consists of 2 medical schools in Xi'an Jiaotong University and 4-5 assistant investigators in project counties. Each investigation team was responsible for the survey of 2-3 project counties in each province (including observation of 5 township hospitals and 20 village clinics in each county, 320 households surveyed, 320 children's physical measurements, and 160 pairs of children And her mother's hemoglobin measurement).
The team leader is responsible for the organization and management of routine investigations. Investigators in each team should cooperate with each other to supervise and inspect the questionnaire.

**Data collection**

Township hospitals and village clinics on-site observation table interview by the investigators observed township hospitals, village clinics and fill out. Investigators also have to go home to conduct surveys of nutrition and health of women and children, physical measurements of children and hemoglobin measurements. County questionnaire filled out by the county health bureau.

**Schedule**
June 15-17, to conducte centralized and unified training in Xi'an.
On June 23, to start the investigation.
The deadline of survey is August 31.

**Quality control**
To ensure the quality, minimize the survey error and establish a comprehensive quality inspection system at every level of the original data of the investigation, we should:
1. On-the-spot investigation, after each visit record, investigators should conduct a comprehensive inspection of the contents to be filled in.

2. The investigation team should investigate the contents of the questionnaire daily check.
3. The investigation team leader on the investigators sent through the review of the questionnaire, carefully verified, before signing the signature.

**Investigation of the required equipment**

1. Supine position measuring device

2. Baby scales
3.Hemoglobin meters, all provided by UNICEF

**Work manual**
A) Investigator duties
1. Obedience to organizational leadership, strict observance of the investigation discipline, the investigation shall not be free to leave their posts
2. Seriously study training materials, survey description and measurement methods, conduct an objective and realistic investigation, fill in a form
3. The investigation of difficult problems, should be promptly reported to the team responsible officers, to discuss the solution
4. After the completion of the questionnaire should be promptly verified, the questionnaire submitted intact

B) Site work guidelines
The key to the success or failure of any survey is the quality of data collection. Therefore, the quality of the work of investigators is of crucial importance.

C) Preparation before the investigation
1. Investigators must receive training, carry out on-the-spot training, read carefully the training materials, especially the anthropometric skills and considerations in measurement.

2. Ask the respondent households (usually two to three days in advance) to contact the respondents, agree on the survey time and place of investigation, and should advise them to return as far as possible and make an appointment for the survey before leaving the home.
3. Ask the investigator to have full preparation for the visit. Since most of the surveys are for mothers and children, some questions (anthropometry) can not be replaced by others, so investigators may need to visit again and can not give up until after repeated hard-won efforts.
4. Before the formal investigation, the organizational investigators conscientiously study and fill in descriptions and various measurement methods, requiring investigators to master the measurement methods and inquiry methods.
5. Investigators should note that any findings from each household are kept confidential.
6. Personnel at all levels of the province, county, township and village levels should be reminded of the questions answered by mothers, village doctors and township hospitals of children

D) Investigation

1. After entering the village to determine the respondent. Village clinics on-site observation of the respondent is the village health clinic is responsible for preventive health care physicians. The respondents to the family questionnaire were mothers with children under 3 years of age and responders to anthropometry were children under 3. Respondents to hemoglobin testing were children under 3 years of age and their mothers.

2. During the investigation, investigators should maintain an objective attitude. Avoid obvious introductory questions, do not talk to respondents about their own views on the proposed questions to be respondents to answer completely, do not subjective guess the respondent's answer, investigators should try to avoid affecting the respondents Answer the result.
3. Investigators should follow all the original design of the questionnaire, asking one by one, do not use their own words to ask questions. The respondent may not interpret the original intention of the problem due to the cultural level or the language barrier, and the investigator may explain it appropriately, for example, dialect may be used.
4. Investigators must according to the questionnaire in the order of the questions, according to the instructions to fill in the form, shall not ask unrelated questions or feel free to change the order of questions to prevent leaks

5. According to the actual situation of the first family questionnaire or village clinics on-site survey.
6. The age of children and mothers in the household questionnaire is an important indicator that requires investigators to be strict enough to obtain accurate information.
7. In the field investigation should promptly check the questionnaire, found that leaks, mistakes, mistakes and timely correction. Human body measurements should be timely control of measurement standards, control of measurement quality. Special attention should be paid to values outside the reference range. Only when it is determined that the measurement is correct, can be filled in and signed to confirm.
8. During the on-the-spot investigation in village clinics, please pay attention to the items (whether pressure cooker, syringe type, disinfection method, delivery package, various records) that the investigators need to examine personally. These items should be checked by themselves and not only by the respondents Answer.
9. In the field investigation, children and mothers asked and measured and children and mothers waiting were arranged in separate rooms. Avoid tips and influences on each other's problems

E) After the investigation

 Check the questionnaire: After completing all the household survey, you must make a complete inspection on the questionnaire you filled in to see if there are any missing items, writing errors, and logic errors. If any doubt is found, the respondent should be asked to repeat the explanation and amend the original record. For each completed questionnaire should check the following items:
1) Check whether each item in the questionnaire is filled in.
2) Check whether there is an inconsistent answer, whether there is a violation of the logic of the error exists.
3) Whether the child's birth date is accurate to the day, and whether the child and mother's birthday use the solar calendar.
Interviewers are cautioned that in order to correct minor mistakes that are obviously due to their own negligence, no unauthorized correction is allowed until they have been revisited

**Special circumstances**
1. Refused to answer: Whether respondents are willing to cooperate depends to a large extent on the first time an investigator gave him / her the impression. It is very important for investigators to make proper self-introduction before starting the investigation, to explain their purpose and nature, and to emphasize the principle of confidentiality. If the respondent said she was unwilling to be investigated, do not immediately decide to refuse the answer, perhaps because of the inappropriate time or place or misunderstanding of the purpose of the investigation. In this case, be patient and explain it again. If the respondent still refuses to cooperate, then he / she can be considered as uncooperative. Replace as required
2. The respondents were not at home: During the investigation, when the interviewee was not at home, the interviewer had to revisit the interview and could not give up until there were no three visits.
3. Other reasons: If the first investigation is not completed due to unexpected interruption of investigation or because the investigator has missed some questions, it should arrange a return visit and complete the survey without repeating the entire investigation

**General requirements**

1.The respondents to this questionnaire are sampling villages of clinics in the sample counties of the project counties. The village clinics surveyed shall be responsible for preventive health care in this village.
2. In the case of each clinic, a questionnaire should be completed by the investigator.
3. The code consists of 8 digits. The first 6 digits are the administrative code of the county and are uniformly provided by the Ministry of Health. The 7th digit is the code of the township. The 8th digit is the code of the village. This code should correspond to the first eight digits of the household questionnaire.
4. According to the survey respondents' responses, the investigators fill in the lines according to the requirements or circle the numbers that match the answers.

5. The codes on the horizontal line on the right side of the table are for computer entry. Fill in by investigator as required. According to the survey respondents' responses, the investigators filled in the corresponding numbers on the horizontal lines of the questions.

6. Jump Tip: Investigators should pay attention to the jump behind the prompts, and asked to ask questions.
7. Multiple choice: Some questions after the option is not the only, investigators do not have to prompt, should make the respondents try to remember, think,
8. The first audit refers to the completion of each table after the investigators on-site review, a comprehensive examination of the contents of the fill.
The second audit refers to the mutual investigation between investigators after the investigation in each village and also on the spot.

**Audit**

**1.** Firstly, please investigators do a good job in the field to check the questionnaire itself is complete, with or without gaps, errors, writing is not clear and so on. Timely questioning and correcting problems found. And sign to confirm.

 2. Secondly, before leaving the scene, the investigators handed over the questionnaires they reviewed to the investigation teammate for review.

 3. Thirdly, after the end of each day's work, ask the interviewer to focus on the questionnaires and measurements of all the surveys conducted today and check whether the tasks are completed correctly and the signature is correct. Record the problems and what you see and hear in the diary of the day
